# Supplementary material for: Pseudoirreversible inhibition elicits persistent efficacy of a sphingosine 1-phosphate receptor 1 antagonist
Source: Nat Commun. 2024 Jul 19;15:5743. doi: 10.1038/s41467-024-49893-8 (PMC11271513; doi:10.1038/s41467-024-49893-8)
Supplement: Supplementary file 3 — Description of Additional Supplementary Files [file 41467_2024_49893_MOESM3_ESM.pdf]

Description of Additional Supplementary Files

File name: Supplementary Movie 1

Description: A typical movie of MetaD simulation for the interaction between *(R)*-KSI-6666 and S1PR1

File name: Supplementary Movie 2

Description: A typical movie of MetaD simulation for the interaction between W146 and S1PR1

File name: Supplementary Movie 3

Description: A typical movie of MetaD simulation for the interaction between *(R)*-KSI-6666 and S1PR1 Val124 mutant
