# Supplementary material for: Pseudoirreversible inhibition elicits persistent efficacy of a sphingosine 1-phosphate receptor 1 antagonist
Source: Nat Commun. 2024 Jul 19;15:5743. doi: 10.1038/s41467-024-49893-8 (PMC11271513; doi:10.1038/s41467-024-49893-8)
Supplement: Supplementary file 7 — Reporting Summary [file 41467_2024_49893_MOESM7_ESM.pdf]

nature portfolio

Corresponding author(s): Taishin Akiyama

Last updated by author(s): May 11, 2024

Reporting Summary

Nature Portfolio wishes to improve the reproducibility of the work that we publish. This form provides structure for consistency and transparency in reporting. For further information on Nature Portfolio policies, see our Editorial Policies and the Editorial Policy Checklist.

Statistics

For all statistical analyses, confirm that the following items are present in the figure legend, table legend, main text, or Methods section.

n/a	Confirmed	
☐	☒	The exact sample size (n) for each experimental group/condition, given as a discrete number and unit of measurement
☐	☒	A statement on whether measurements were taken from distinct samples or whether the same sample was measured repeatedly
☐	☒	The statistical test(s) used AND whether they are one- or two-sided<br>*Only common tests should be described solely by name; describe more complex techniques in the Methods section.*
☒	☐	A description of all covariates tested
☐	☒	A description of any assumptions or corrections, such as tests of normality and adjustment for multiple comparisons
☐	☒	A full description of the statistical parameters including central tendency (e.g. means) or other basic estimates (e.g. regression coefficient) AND variation (e.g. standard deviation) or associated estimates of uncertainty (e.g. confidence intervals)
☐	☒	For null hypothesis testing, the test statistic (e.g. F, t, r) with confidence intervals, effect sizes, degrees of freedom and P value noted<br>*Give P values as exact values whenever suitable.*
☒	☐	For Bayesian analysis, information on the choice of priors and Markov chain Monte Carlo settings
☒	☐	For hierarchical and complex designs, identification of the appropriate level for tests and full reporting of outcomes
☒	☐	Estimates of effect sizes (e.g. Cohen's d, Pearson's r), indicating how they were calculated

Our web collection on statistics for biologists contains articles on many of the points above.

Software and code

Policy information about availability of computer code

Data collection	Leukocytes and lymphocytes in blood were counted by KX-21NV (Sysmex), ProCyte Dx (IDEXX) and ADVIA120 (Siemens). Concentrations of compounds were measured by Q Exactive (Thermo Fisher Scientific). Fluorescence or luminescence signals were measured by PHERAstar FSX (BMG Labtech). The increase in cellular calcium level was evaluated using an FDSS 7000 (Hamamatsu Photonics). Heart rate was monitored and recorded by a PowerLab 8/35 instrument (AD Instruments) and LabChart software (AD Instruments). Respiratory rate and Penh were recorded by FinePointe Systems (Buxco). Flow cytometry data were collected by a FACS Canto (BD) and SA3800 (SONY). Real-time PCR was performed using a 7500 Fast Real-Time PCR System (Applied Biosystems). Optical density was measured for ELISA by a SpectraMax 190 (Molecular Devices).
Data analysis	Microsoft Excel for Microsoft 365 was used for data analysis. Statistical analysis and nonlinear regression analysis were performed by GraphPad Prism 6 software, DescTools version 0.99.54 and multcomp version 1.4-25 packages in R version 4.3.1. Data analysis of heart rate was performed by a LabChart software (AD Instruments). Data processing for LC-HRMS was performed using Quan Browser software version2.2 (Thermo Fisher Scientific). The pharmacokinetic parameters were calculated using WinNonlin 6.1 software (Pharsight). Flow cytometry data were analyzed by a FlowJo version 10 (BD). Optical density analysis was performed by a SoftMaxPro Software v5.4.1 (Molecular Devices). For molecular docking analysis, Maestro (Schrödinger LLC), Glide (Schrödinger LLC) and Prime (Schrödinger LLC) were used. For molecular dynamics analysis, Maestro and Desmond version 2.3 (Schrödinger LLC) were used. PLIF analyses were performed using a MOE 2020.0901 (Chemical Computing Group Inc.). The MM-GBSA protocol (Schrödinger LLC) was used to calculate the binding free energy of ligands. For fragment molecular orbital analysis, Impref module (Schrödinger LLC), ABINIT-MP and MIZUHO/BioStation viewer were used.

For manuscripts utilizing custom algorithms or software that are central to the research but not yet described in published literature, software must be made available to editors and reviewers. We strongly encourage code deposition in a community repository (e.g. GitHub). See the Nature Portfolio guidelines for submitting code & software for further information.

Data

Policy information about availability of data

All manuscripts must include a data availability statement. This statement should provide the following information, where applicable:

- Accession codes, unique identifiers, or web links for publicly available datasets
- A description of any restrictions on data availability
- For clinical datasets or third party data, please ensure that the statement adheres to our policy

Source data are provided within this paper. The MD simulation data including input files and final output configurations have been deposited to GitHub depository (https://github.com/CHEMINFO-tsukuba/Nat.Comm.2024.git).Photo image data generated in this study have been deposited in the Zenodo database [10.5281/zenodo.11260928].

Research involving human participants, their data, or biological material

Policy information about studies with human participants or human data. See also policy information about sex, gender (identity/presentation), and sexual orientation and race, ethnicity and racism.

Reporting on sex and gender	n/a
Reporting on race, ethnicity, or other socially relevant groupings	n/a
Population characteristics	n/a
Recruitment	n/a
Ethics oversight	n/a

Note that full information on the approval of the study protocol must also be provided in the manuscript.

Field-specific reporting

Please select the one below that is the best fit for your research. If you are not sure, read the appropriate sections before making your selection.

☒ Life sciences ☐ Behavioural & social sciences ☐ Ecological, evolutionary & environmental sciences

For a reference copy of the document with all sections, see nature.com/documents/nr-reporting-summary-flat.pdf

Life sciences study design

All studies must disclose on these points even when the disclosure is negative.

Sample size	Sample size was not determined by statistical methods but was determined based on common practice to reach statistical significance compared to control.
Data exclusions	No data was excluded.
Replication	At least three independent experiments were performed. Number of experiments are indicated in figure legends.
Randomization	For in vivo experiment, animals were randomly assigned to each group.
Blinding	The histopathological assessment was performed without blinding. Every sample was scored by several board-certified veterinary pathologists and the scoring obtained was furthermore double-checked by other experts.

Reporting for specific materials, systems and methods

We require information from authors about some types of materials, experimental systems and methods used in many studies. Here, indicate whether each material, system or method listed is relevant to your study. If you are not sure if a list item applies to your research, read the appropriate section before selecting a response.

Materials & experimental systems

n/a	Involved in the study
☐	☒ Antibodies
☐	☒ Eukaryotic cell lines
☒	☐ Palaeontology and archaeology
☐	☒ Animals and other organisms
☒	☐ Clinical data
☒	☐ Dual use research of concern
☒	☐ Plants

Methods

n/a	Involved in the study
☒	☐ ChIP-seq
☐	☒ Flow cytometry
☒	☐ MRI-based neuroimaging

Antibodies

Antibodies used	Anti-mouse CD19-PE (BD 557399, https://www.bdbiosciences.com/ja-jp/products/reagents/flow-cytometry-reagents/research-reagents/single-color-antibodies-ruo/pe-rat-anti-mouse-cd19.557399, 1:400), CD4-PECy7 (BioLegend 100528, https://www.biolegend.com/ja-jp/products/pe-cyanine7-anti-mouse-cd4-antibody-1932, 1:400), CD8-APCCy7 (BioLegend 100714, https://www.biolegend.com/ja-jp/products/apc-cyanine7-anti-mouse-cd8a-antibody-2269,1:400), anti-CD16/32 (BioLegend 101302, https://www.biolegend.com/ja-jp/products/purified-anti-mouse-cd16-32-antibody-190, 1:200) and CD45RB-FITC (BioLegend 103305, https://www.biolegend.com/en-us/products/fitc-anti-mouse-cd45rb-antibody-222?GroupID=BLG6845, 1:364) antibodies were used. Anti-FITC MicroBeads (Miltenyi Biotec 130-048-701, https://www.miltenyibiotec.com/US-en/products/anti-fitc-microbeads.html#130-048-701, 1:10) and anti-human S1PR1-PE antibody (R&D Systems FAB2016P, https://www.rndsystems.com/products/human-s1p1-edg-1-pe-conjugated-antibody-218713_fab2016p, 1:3.5) were also used.
Validation	All antibodies are commercially available. We followed the statements on the manufacturer's websites.

Eukaryotic cell lines

Policy information about cell lines and Sex and Gender in Research

Cell line source(s)	HEK293 (RIKEN BRC) and CHO-K1 (ECACC) were used.
Authentication	Cell lines obtained from the distributors were appropriately cultured, cryopreserved and used in our facility.
Mycoplasma contamination	The cell lines were not tested for mycoplasma contamination but cells obtained from the distributors were appropriately cultured, cryopreserved and used in our facility.
Commonly misidentified lines (See ICLAC register)	None of misidentified cell lines were used.

Animals and other research organisms

Policy information about studies involving animals; ARRIVE guidelines recommended for reporting animal research, and Sex and Gender in Research

Laboratory animals	Information about the laboratory animals (strains, distributors, age, sex, breeding conditions) used are described in the material and methods section. BALB/cA mice (CLEA Japan and Jackson Laboratory Japan), BALB/cCr mice (Japan SLC), C57BL/6JJcl mice (CLEA Japan), C.B-17 SCID mice (Jackson Laboratory Japan), Sprague-Dawley (SD) rats (Japan SLC and Jackson Laboratory Japan), Dark Agouti (DA) rats (Japan SLC), Hartley guinea pigs (Japan SLC), and Cynomolgus monkeys (Del Mundo Trading and SICONBREC Inc.) . Breeding rooms were kept 12 hr dark/light cycle; temperature: 21-25°C in RIKEN, 20-26°C in Kissei Pharmaceutical Co., Ltd and 22-28°C in Ina Research Inc.; humidity: 40-60% in RIKEN, 40-70% in Kissei Pharmaceutical Co., Ltd and 40-80% in Ina Research Inc.
Wild animals	No wild animals were involved.
Reporting on sex	Basically male animals were selected to minimize the effect of sexual cycle but in some studies female mice were used because adult male mice often fight and hurt each other in the cages.
Field-collected samples	No field-collected samples were involved.
Ethics oversight	Experiments were performed in accordance with the protocols approved by the Laboratory Animal Committee of Kissei Pharmaceutical Co., Ltd. or by the Institutional Animal Care and Use Committee, or in accordance with the Guidelines of the Institutional Animal Care and Use Committee of RIKEN, Yokohama Branch (2018-075). Experiments performed in Ina Research Inc. followed the protocols approved by the Institutional Animal Care and Use Committee.

Note that full information on the approval of the study protocol must also be provided in the manuscript.

Flow Cytometry

Plots

Confirm that:

☒ The axis labels state the marker and fluorochrome used (e.g. CD4-FITC).

☒ The axis scales are clearly visible. Include numbers along axes only for bottom left plot of group (a 'group' is an analysis of identical markers).

☒ All plots are contour plots with outliers or pseudocolor plots.

☒ A numerical value for number of cells or percentage (with statistics) is provided.

Methodology

Sample preparation	Whole blood from mice was collected into tubes pretreated with EDTA. After the lysis of erythrocytes by using RBC Lysis Buffer, cell suspensions were stained with labeled-antibodies. HEK293 cells stably expressing human S1PR1 were collected after the wash by phosphate-buffered saline and detachment using Versene.
Instrument	FACSCanto (BD), spectral cell analyzer SA3800 (SONY)
Software	Flowjo v.10
Cell population abundance	n/a
Gating strategy	Live lymphocytes in blood were determined by FSC/SSC, 7-AAD, and lymphocyte gates. Live HEK293 cells stably expressing human S1PR1 were determined by FSC/SSC and 7-AAD.

☐ Tick this box to confirm that a figure exemplifying the gating strategy is provided in the Supplementary Information.
